# Ghadeer-speech-crowd-corpus: Speech dataset

**DOI:** 10.1016/j.dib.2024.111201

**Published:** 2024-12-09

**Authors:** Ghadeer Qasim Ali, Husam Ali Abdulmohsin

**Affiliations:** Computer Science Department, College of Science, University of Baghdad, Iraq

**Keywords:** Arabic phrase, English phrase, Speech recognition, Low-resource languages

## Abstract

The availability of raw data is a considerable challenge across most branches of science. In the absence of data, neither experiments can be conducted nor development can be undertaken. Despite their importance, raw data are still lacking across many scientific fields. A literature survey conducted at the beginning of our study indicated a significant lack of Arabic speech datasets. Therefore, this study aims to address this problem by proposing a new Arabic and English dataset called Ghadeer-Speech-Crowd-Corpus. This dataset was designed to target more than one branch of speech-processing applications, such as crowd speaker identification, speech synthesis (text-to-speech), and speech recognition (speech-to-text). Speech samples were recorded over three months from 210 Iraqi Arab citizens living in different parts of Iraq and included more than one accent. The proposed dataset was fully balanced with respect to sex and recordings (same number of Arabic and English recordings). Additionally, it is a mono dataset and contains 15,626 audio samples recorded at a sampling rate of 44,100 Hz, 16-bit depth, and bit rate of 705.6 kb/s. The recordings were conducted at the Academy for Media Training of the College of Media, University of Baghdad.

Specifications Table

**Table:** 

Subject	Computer science, signal processing.
Specific subject area	Speech recognition in different environments.
Type of data	Audio and text.
Data collection	The data-collection process was divided into two parts. In the first part (solo), each participant was asked to speak nine sentences each in Arabic and English; thus, 18 samples were collected from each participant. In the second part (crowd), the 210 participants were divided into groups of 2, 3, 4, and 5, and each participant could participate in more than one group. Each group simultaneously recorded the 18 samples through the same microphone, as shown in figure 2. The following tools were used in this process: • Adobe Audition 1.5, Tascam mixer, and Sennheiser microphone (E825-S). • Audacity and free mp3 cutting for audio processing. • Window 11.
Data-source location	Institution: University of Baghdad. City/town/region: Baghdad/Al-Jadriya. Country: Iraq.
Data accessibility	Repository name: Mendeley Data Data identification number: 10.17632/mkhjfnzty5.1 Direct URL to data: https://data.mendeley.com/datasets/mkhjfnzty5/1

## 1 Value of the Data

• It can help organizations and individuals involved in the field of machine/deep learning develop speech-based products and services.
• It comprises raw data that can be used in noise-reduction applications.
• It provides insights into the Arabic and English accents spoken by Iraqi citizens.
• It includes biometrics, such as age [1], sex [2], height, and weight, that can be used in biometrics-based speech evaluation and prediction applications.
• It can be employed in speaker identification and authentication applications because the audio coding refers to the individuals who participated in the recording process.
• As it includes the text used in all audio clips, it can be used in speech recognition (speech-to-text) applications.
• It is the first dataset that contains solo and crowd recordings of the same individuals.
• Although many speech datasets, such as LibriSpeech [3], TIMIT [4], Wall Street Journal [5], VoxCeleb [6], Ar-DAD [7], MASC [8], as well as a novel dataset for Arabic Speech Recognition recorded by Tamazight Speakers are available [9], none of them contain both Arabic and English recordings of the same individuals.

## 2 Background

The primary motivation behind designing and generating this dataset was the fulfillment of our master's degree. Our master's project focused on crowd identification. However, during our literature survey, we noticed a shortcoming in the datasets, recording three, four, and five speakers of Arabic and English.

## 3 Data Description

**Fig. 1:**
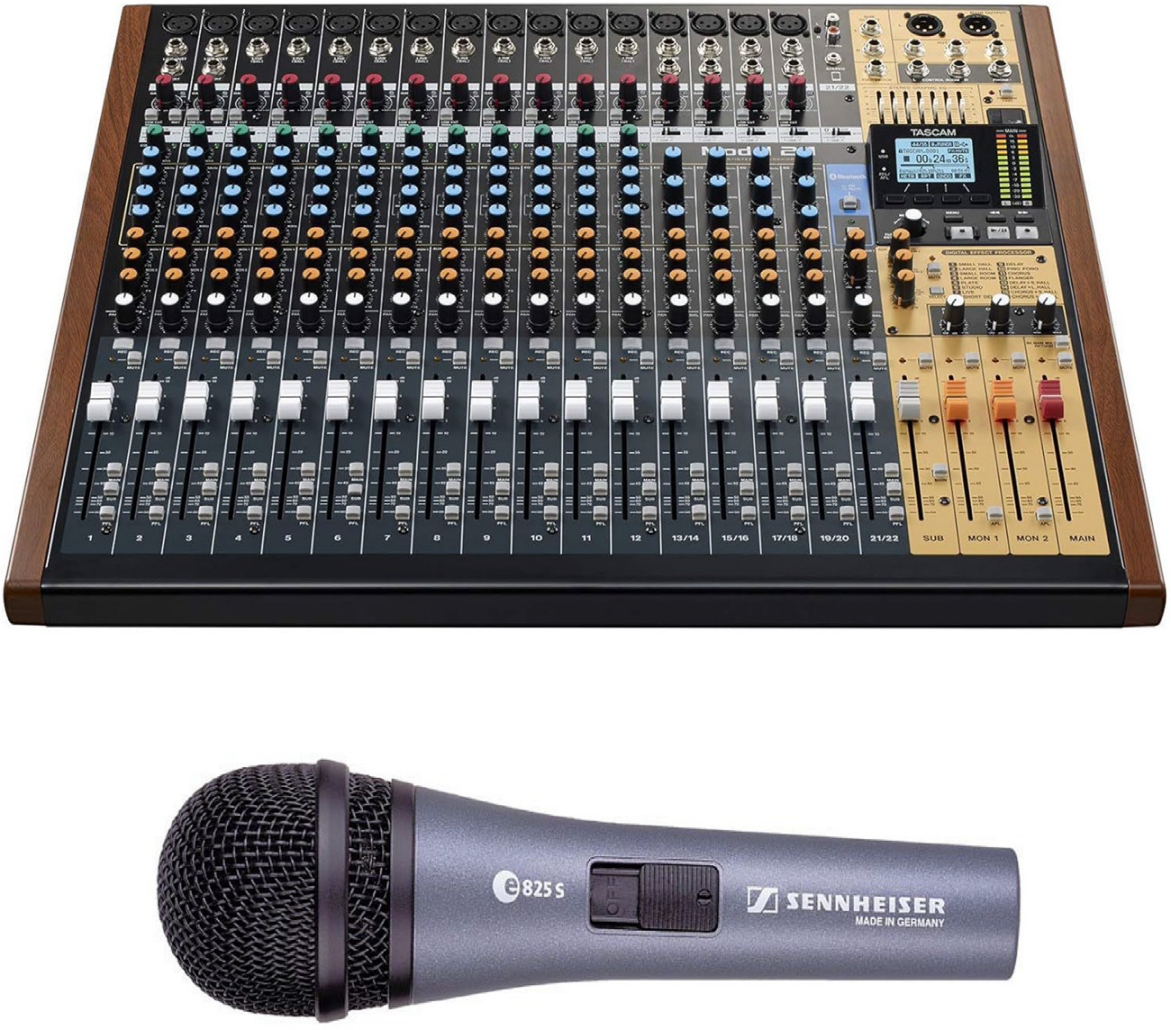
The recording device used Mixer, Microphone.

All participants provided written informed consent for including their recordings in the dataset. This file presents an example of the obtained consent, including the use of their recording in both languages and participant characteristics, such as age, sex, height, and weight (Fig. 1).

**Fig. 2:**
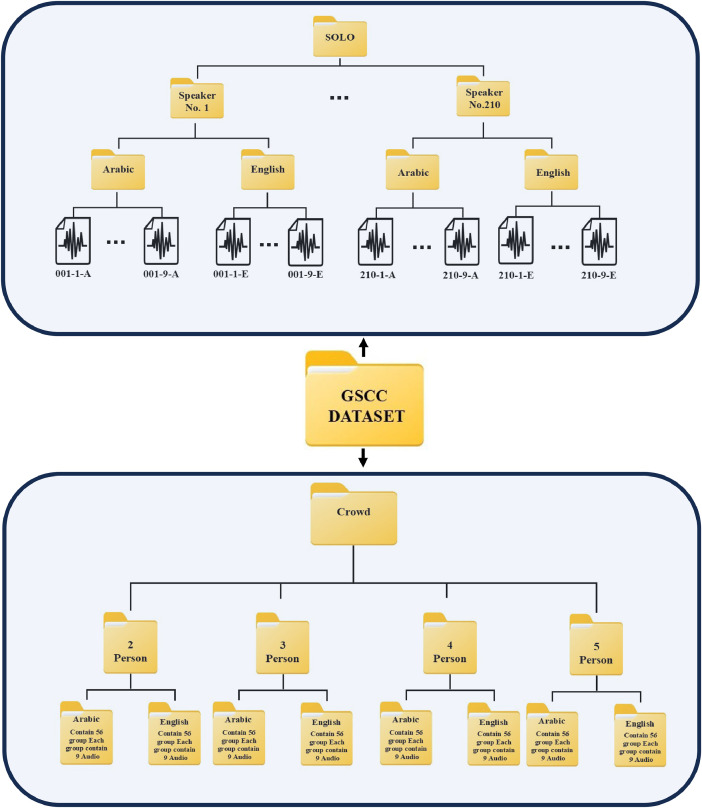
Architecture of the dataset.

The Ghadeer-Speech-Crowd-Corpus (GSCC) dataset contains two types of recordings—solo and crowd—stored in separate folders in wave and mp3 formats. The crowd speech folder contains four subfolders, each with a different number of crowds. The crowd recordings included two, three, four, and five individuals. Each crowd subfolder contains two subfolders: one each for Arabic and English. There were 56 groups who spoke 9 sentences in each language, resulting in 504 audio clips. The second folder contains 210 folders comprising solo recordings, with each folder representing one speaker. Additionally, each speaker folder contains two folders: one for each language. Each speaker recorded nine audio clips in each language. Upon downloading and preparing the dataset, the training process is structured as follows: Internal data splitting, also known as Cross-Validation, involves dividing the 9 available sentences for each speaker into 80 % for training (7 sentences) and 20 % for testing (2 sentences). This methodology enhances the modelʼs accuracy by ensuring comprehensive exposure to all data during the training phase. For the recordings in Arabic and English, two approaches can be employed, either developing separate models for each language—one dedicated to Arabic and another to English. In the context of Speaker Recognition model training, the recordings of each speaker are treated as distinct classes, facilitating the modelʼs ability to differentiate among individual speakers. Additionally, group recordings can be utilized to further improve model performance in scenarios involving simultaneous recordings of multiple speakers, thereby enabling the effective identification and separation of distinct vocal inputs. The dataset is organized in a structured manner, with separate folders for individual and group recordings. The architecture of the GSCC is shown in Fig. 2.

## 4 Experimental Design, Materials and Methods

The data-collection and recording processes are detailed in the following sections.

### 4.1 Study area

The data were collected at the University of Baghdad - College of Media. Different groups of students from various colleges at the University of Baghdad participated in the collection. The recordings were collected at the Academy for Media Training of the College of Media at the University of Baghdad. All data were recorded using high-quality materials, as listed in the specifications table.

### 4.2 Participants

In this study, 210 healthy speakers (105 females and 105 males; age: 18–45 years) voluntarily participated in the data-collection process and provided written informed consent. Speakers numbered 1–105 were females, whereas 106–210 were males.

The number of volunteers who participated in this work, and whose pronunciation accuracy was verified, totaled 210. A total of 210 participants were chosen to represent a diverse range of speakers in both Arabic and English. The objective behind selecting this number was to ensure a wide variety of voices and accents, which is essential for ensuring accurate speaker recognition across different vocal characteristics. The number 210 strikes a balance between the need for a large dataset and the computational constraints of processing and analyzing the data effectively. The selection criteria for these 210 individuals were based on several factors:

(1) Clear and accurate pronunciation.
(2) Maintaining a balance between male and female participants.
(3) Ensuring diversity across different age groups.

This approach guarantees a comprehensive dataset that reflects a broad spectrum of vocal characteristics, further enhancing the model's ability to generalize across various demographic groups.

They all belonged to the Iraqi population and were selected through a rigorous survey and questionnaire, which was direct, unbiased, and unambiguous. The following questions were included:

• Can you spare three hours to help us create a dataset of voice recordings for scientific research?
• Do you have any reservations regarding going to a studio with us for one hour to conduct the recording?
• What is your name?
• What is your age, weight, and height?
• Do you have problems with pronouncing certain words?
• Do you have problems with certain characters?
• Do you have problems recording your voice?
• Do you have any reservations with sharing your voice online for scientific research?
• Do you wear orthodontics?
• Do you have claustrophobia (fear of enclosed spaces)?
• Do you have reservations with being close to girls or boys while recording (because you will be close to each other while recording as only one microphone will be used)?

### 4.3 Experimental procedure

The recording material contained 18 phonetically balanced sentences (nine each in Arabic and English). The Arabic sentences were selected based on surveys conducted by other researchers [10,11]. The sentences were selected randomly to ensure the pronunciation of all letters in both languages, ensuring phonetic diversity. This approach helps capture a broad range of speech patterns and phonetic features for each speaker as shown in Table 1. Each participant was asked to read all 18 sentences to generate 18 solo recordings.

**Table 1:** GSCC sentences.

Additionally, each participant participated in five crowd recordings through five different groups, which comprised two, three, four, or five participants. Each group provided 18 recordings (nine each in Arabic and English). While recording crowd samples, each participant was asked to narrate a different sentence. The lengths of the recordings (solo and crowd) ranged from 3 to 6 s, each participant took approximately 4 min in record their individual sentences. Each group of the 56 groups took approximately 4 min in recording nine sentences, which means 224 recording minutes for each of the two, three, four and five crowded recordings. That makes the total number of crowded recording minutes is 896 min.

### 4.4 Data collection

This section describes the different stages of selecting participant selection, processing recordings, and unifying the collected data.

Stage 1. Participant Selection

The participant selection for this dataset followed the fixed-design regulations. Generating a gender-balanced dataset was one of the objectives of this study. We ensured that the pronunciations of all participants were correct, and a second check was performed while adhering to the recordings. Some additional regulations were imposed, such as females were required to tie their hair and the participants could not wear bracelets or any other jewelry that could generate noise. Additionally, they were asked to turn off all digital devices during the recording process.

Stage 2. Raw Data Preprocessing

During the recording process, the participants read 18 sentences (Arabic and English) together as one paragraph. Subsequently, we cut the audio into 18 clips (nine each for Arabic and English), one for each of the 18 sentences, to simplify and accelerate the process. The cutting process was performed using a free mp3 cutter [12] and Audacity [13].

Stage 3. Coding

**Fig. 3:**
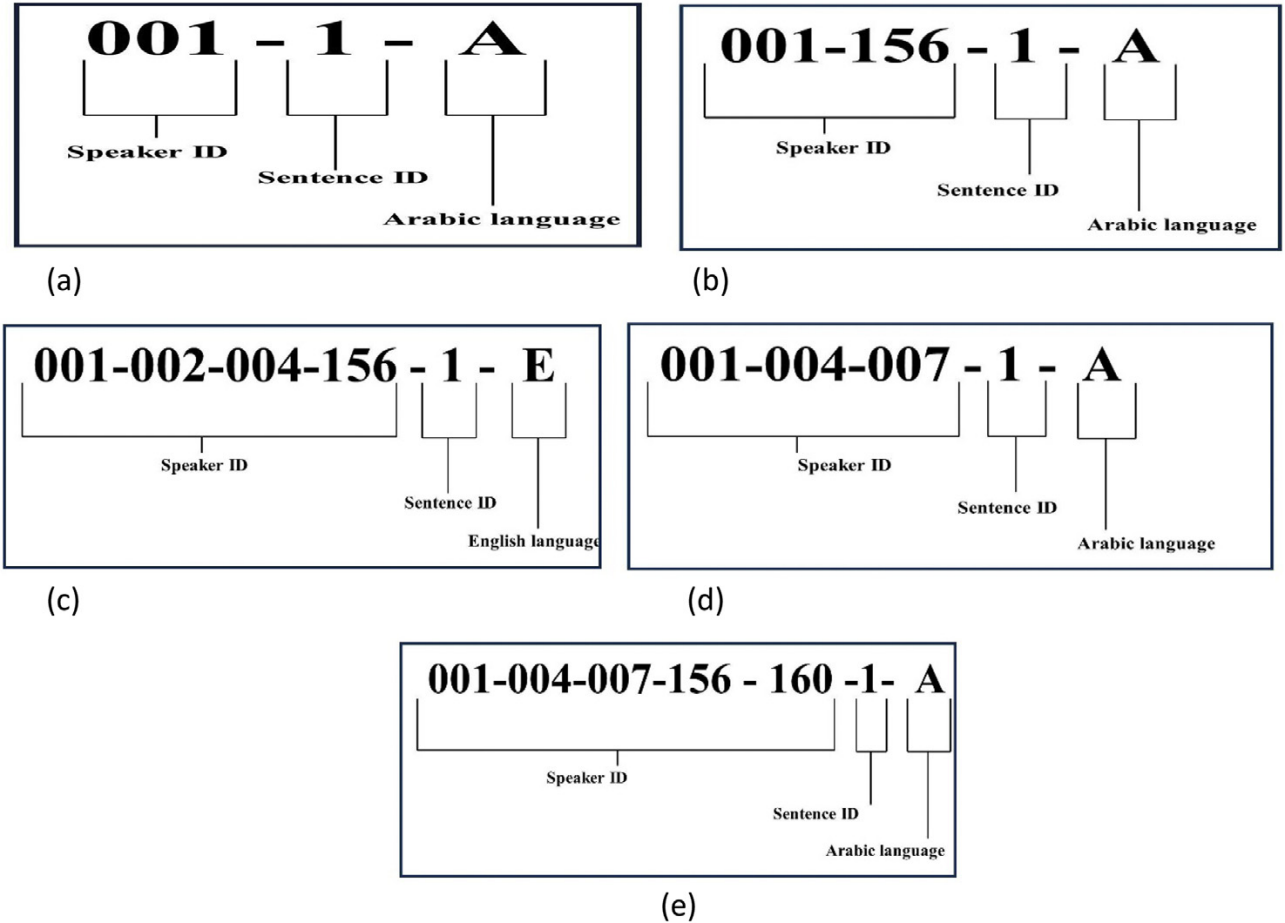
Coding system design. (a) solo speaker, (b) 2 crowd speakers, (c) 3 crowd speakers, (d) 4 crowd speakers, (e) 5 crowd speakers.

To simplify the dataset, each file was coded, which was also useful for creating training and test sets. The coding-system design is shown in Fig. 3. The code starts with the speaker number, followed by an underscore, the number of sentences recorded, and the language used, indicated by “A” for Arabic and “E” for English (Table 2).

**Table 2:** Additional Information for dataset.

Additional Information	
Participants	210 (105 female and 105 male) the labeling from 001 to 105 represent female and from 106 to 210 male
Data	Demographic data, physical data Arabic audio recordings, English audio recordings, Arabic texts, English texts
Characteristics	Large and diverse dataset, high-quality recordings, reliable dataset
Usage	Can be used by researchers and developers working on a variety of projects related to Arabic and English language
Size	2.27 GB (15,626 Files, 1180 Folders)
Completion time	Three months, nearly 30 h for record and 30 h for collect student.

## Limitations

‘Not applicable’.

## Ethics Statement

Informed consent to release the speech-audio recordings was acquired from each participating actor. Participants were informed that participation was voluntary and that they were free to leave or pause the speech recordings at any time. All data collected from the voluntarily participating actors were anonymized after the full data collection process. It was ensured that any information submitted would be treated confidentially and in an anonymous manner. Each participant has approved the samples post-recording by filling and signing an information form. No ethical approval was required.

## Credit Author Statement

**Ghadeer Qasim:** software, validation, writing-reviewing and editing. **Husam Ali:** supervision, data curation, writing, original draft preparation.

## Data Availability

Mendeley DataGhadeer-Speech-Crowd-Corpus (Original data).
